# Mobile mental health: a challenging research agenda

**DOI:** 10.3402/ejpt.v6.27882

**Published:** 2015-05-19

**Authors:** Miranda Olff

**Affiliations:** 1 Department of Psychiatry, Academic Medical Centre, University of Amsterdam, Amsterdam The Netherlands; 2 Arq Psychotrauma Expert Group, Diemen, The Netherlands

**Keywords:** e-Health, mobile health, m-Health, smartphone, mobile apps/applications, global health, trauma, PTSD, depression, internet intervention

## Abstract

The field of mobile health (“m-Health”) is evolving rapidly and there is an explosive growth of psychological tools on the market. Exciting high-tech developments may identify symptoms, help individuals manage their own mental health, encourage help seeking, and provide both preventive and therapeutic interventions. This development has the potential to be an efficient cost-effective approach reducing waiting lists and serving a considerable portion of people globally (“g-Health”). However, few of the mobile applications (apps) have been rigorously evaluated. There is little information on how valid screening and assessment tools are, which of the mobile intervention apps are effective, or how well mobile apps compare to face-to-face treatments. But how feasible is rigorous scientific evaluation with the rising demands from policy makers, business partners, and users for their quick release? In this paper, developments in m-Health tools—targeting screening, assessment, prevention, and treatment—are reviewed with examples from the field of trauma and posttraumatic stress disorder. The academic challenges in developing and evaluating m-Health tools are being addressed. Evidence-based guidance is needed on appropriate research designs that may overcome some of the public and ethical challenges (e.g., equity, availability) and the market-driven wish to have mobile apps in the “App Store” yesterday rather than tomorrow.

Since about 1990, the internet has been booming. It offered new possibilities for electronic communication to improve health and health care delivery (“e-Health”). Mobile health (m-Health), as a form of e-Health, focuses on the development of smartphone applications (apps) to improve medical or mental health care. Mobile platforms are more and more available, more user-friendly, have more attractive designs, and contain increasingly complex computational models and fancy technologies. The use of mobile apps in the field of mental health care is growing rapidly, and m-Health has already been presented as a new frontier for delivering mental health treatment (Price et al., 2014).

Mobile apps may be used to provide access to relevant information or psychoeducation (when and where needed), as well as helping individuals to self-identify symptoms, offering screening and assessment tools, helping people manage their own mental health and wellness, identifying the need for treatment, encouraging help seeking, and providing direct interventions. Once in treatment they may help to engage the patient and maximize retention. m-Health may also be used to inform the practitioner about a patient's status, for example, real-time monitoring of patient symptoms (“ecological momentary assessment”) or, where treatment is undertaken at a distance, as part of the ongoing evaluation of response telemonitoring (telemedicine). Mobile devices can be used to be informed about health data both at individual patient level as well as at an aggregated level (“big data”), for instance, after disasters. New developments are becoming available that use this type of data to direct policy makers.

With m-Health there is great potential to increase availability and equitable distribution and resources for mental health care, especially from a global perspective. Although the term g-Health is sometimes being used for serious gaming (used to train or educate users on a “serious” issue), or for gamification (turning the learning process into a game), it also refers to global health and, in particular, to the use of health-related mobile apps worldwide. Although effective psychotherapies are already available for mental health disorders, many patients are unable to access appropriate treatment. m-Health applications can be an efficient way to serve a considerable proportion of people all over the world, thus potentially addressing unmet mental health care needs. They also offer a wonderful opportunity to reach people in low- and middle-income countries where fewer clinicians may be available for diagnosis, treatment, and management of disease but where a large proportion of people seem to possess a smartphone. In Table 1, these and other advantages of m-Health are listed.

**Table 1:** Advantages of m-Health

*Capacity*—Mental health care institutions often do not have the capacity to provide everyone with the care they need because it is time-consuming and costly or people live far away. m-Health tools may support therapy and increase efficacy and efficiency or reduce wait lists.
*24/7 availability—*Individuals carry their smartphones with them throughout the day (and night for some) and can use mobile applications whenever, wherever they want. This may be especially helpful for tools that prompt individuals to take their medications in time, do their exercises, and monitor their health status.
*Equity—*In areas with resource scarcity m-Health may increase access to mental health care and contribute to fairness in the distribution of mental health care resources.
*Immediate support*—For individuals with symptoms (e.g., anxiety) which are triggered by external reminders in the individual's environment mobile apps could offer immediate support by providing tools and exercises that help manage symptoms. Patients may use the apps outside the 1-hour weekly therapy session to support treatment.
*Anonymity*—m-Health tools provide anonymity and non-stigmatizing support to people searching mental health advice or treatment.
*Tailored approach—*m-Health can be tailored to the individual, addressing personal needs and “remembering” these. Users are in control over what, when, and where to use the app (self-paced), have access independent of a health care provider, and a variety in modalities exist (text-based, interactive, multiple media) to match individual learning styles.
*Linking into other systems*—m-Health can be linked to wearables, other apps, or features. Native apps which are built for a specific platform (e.g., iOS or Android) may be more advanced in having access to specific hardware features (e.g., camera, microphone, GPS, calendar) as opposed to a web-based app which is hosted on the web. Advantage of the latter is that these can be accessed from any a browser on any device with internet access and access to other features is increasing.
*Lower cost*—Widening access to psychological interventions with m-Health as a relatively light intervention is potentially cost-effective compared to traditional interventions.

Mobile apps are being adopted (and also replaced) almost as soon as they have been developed. There is an explosive growth of mobile apps and other e-Health tools, in a field being increasingly dominated by commercial parties. Some apps are free, some have fees, but it is not always clear which ones are valid, reliable, or provide effective interventions. Individuals searching for a mental health app have a range of choices in the app stores (e.g., Shen et al., 2015). Few apps have been rigorously evaluated and the scientific evidence base is clearly running behind the market.

In the remainder of this paper m-Health developments for traumatic stress will be presented as well as the challenges and associated research questions to be addressed.

## m-Health developments for traumatic stress

### Screening and assessment of traumatic stress

Self-tests for almost any disorder can be found on the internet, often with questionable quality. However, initiatives like PROMIS (www.nihpromis.org) aim to provide reliable and valid assessment tools to measure patient-reported health status. Information on valid and reliable traumatic stress tools can be found on websites of traumatic stress societies (e.g., www.ISTSS.org) or specialized institutes (e.g., www.ptsd.va.gov).

Interesting computerized approaches to the assessment of childhood trauma within a relational-socioecological framework have also been developed (Frewen et al., 2013). As yet, however, few validated mobile apps exist for screening and assessment of posttraumatic stress reactions.

Within an EU funded “Knowledge platform for prevention and self-help in mental healthcare” we are currently testing Smart Assessment on your Mobile (SAM), both in high-risk populations, such as the police, and in patients with posttraumatic stress disorder (PTSD). SAM is based on valid, reliable, and freely accessible tools and assesses both resilient and a wide range of (traumatic) stress responses, as well as risk and protective factors. It is smart in the sense that it is tailored to an individual reducing the amount of questions (e.g., it is gender sensitive, no further depression items are administered when initial screening questions are all negative for depression, and no questions on peritraumatic responses will be asked if the event happened long ago). Validation research is ongoing (Van der Meer, 2014).

One of the beauties of smartphones is that they allow for Ecological Momentary Assessments (EMA) (or experienced sampling methods) with real-time assessment of symptoms or behavior. A brief EMA app for Stress and Trauma (STEMA) has been proposed for assessing posttraumatic reactions shortly after trauma, looking specifically, for example, at posttraumatic sleep quality, hyperarousal, and traumatic recall (Olff & Shalev, in prep).

### Prevention of traumatic stress

Regarding the prevention of traumatic stress, there is still little consensus on how to deliver effective early interventions and how to reach all people in need of these interventions in time although recent new exposure-based (Rothbaum et al., 2012) and medication-based (Delahanty et al., 2013; Frijling et al., 2012) approaches show promise. Interesting developments are studies showing that playing the game Tetris shortly after trauma may prevent intrusions of traumatic memories (Holmes, James, Coode-Bate, & Deeprose, 2009). Although there is the potential of reaching many, for instance, in the aftermath of disasters or after road traffic accidents, there are very few e- or m-Health evidence-based preventive tools to date.

An example of an IT-based system in development, containing tools needed to plan, conduct, and evaluate psychosocial support interventions in the context of disasters for psychosocial crisis managers and mental health experts, is the Operational Guidance System (OGS) (see “Operationalising Psychosocial Support in Crisis,” www.opsic.eu). It is based on sound scientific review of existing guidelines and best practice studies in order to provide reliable methods and tools to relevant target groups, types, and phases of emergencies.

One of the few preventive self-guided internet-based interventions is Trauma TIPS, developed to prevent the onset of PTSD symptoms in injury patients (Mouthaan et al., 2013). However, uptake was low and only a significant decrease in PTSD symptoms was found in a subgroup of patients with severe initial symptoms. This type of intervention may be indicated only for individuals with high initial symptoms which may be detected with early screening, for example, by a mobile app.

With regard to mobile apps “PFA Mobile” is a preventive mobile app developed for responders who provide Psychological First Aid (PFA), an evidence-informed approach for assisting individuals in the aftermath of disaster or emergencies (Kuhn, Eftekhari, et al., 2014). The app is not intended to replace PFA training and efficacy still needs to be shown.

### Effective interventions and m-Health

Effective treatments for PTSD include trauma-focused psychotherapies like cognitive processing therapy, cognitive behavioral therapy (CBT), or eye movement desensitization and reprocessing (EMDR) (e.g., Bisson, 2013; Bisson, Roberts, Andrew, Cooper, & Lewis, 2013; Forbes et al., 2010). Mobile apps and internet interventions are often based on these established evidence-based psychotherapies (e.g., Cuijpers, Van Straten, & Andersson, 2008; Mouthaan, Sijbrandij, Reitsma, Gersons, & Olff, 2011).

Efficacy has been shown for e-Health interventions for anxiety and depression (Cuijpers et al., 2008; Kaltenthaler et al., 2006) and also for post-disaster mental health (Ruggiero et al., 2012). For depression, many mobile apps are already on the market (Shen et al., 2015) and other promising new interventions, making use of mobile devices, are currently being studied (e.g., Bockting et al., 2011; Warmerdam et al., 2012).

There is less m-Health research for PTSD compared to other mental health disorders (Lewis, Pearce, & Bisson, 2012). Preliminary support exists for the use of specific web-based treatments in reducing posttraumatic stress symptoms (Bolton & Dorstyn, 2015; Ruzek, Weingardt, Kuhn, & Hoffman, 2011). This type of intervention is also showing promise in research undertaken in non-Western countries like China (Wang & Maercker, 2014). Therapist-assisted web-based interventions for trauma survivors have been shown to be effective, but may require significant input from the health care provider (e.g., Interapy) (Knaevelsrud & Maercker, 2007; Lange et al., 2003).

One of the most well-known mobile apps in the field of traumatic stress is “PTSD Coach,” developed by the Veterans Affairs National Center for PTSD in the United States (Kuhn, Greene, et al., 2014). PTSD Coach is a mobile app designed to help individuals who have PTSD symptoms better understand and self-manage their symptoms. PTSD Coach is based on evidence-based CBT principles and can be used both as a stand-alone app, as well as a supportive app during therapy. It consists of psychoeducation, self-assessment, information about referral and treatment centers, specific tools, and CBT-based exercises to reduce negative trauma-related cognitions, and tools to strengthen social support and psychological resilience. PTSD Coach has been translated into several languages and is being used worldwide (with 50,000 downloads yearly). Kuhn, Greene, et al. (2014) performed a qualitative feasibility study on PTSD Coach and found it to be positively evaluated by American veterans with posttraumatic stress symptoms on acceptability and helpfulness in dealing with symptoms. Studies on the efficacy of PTSD Coach in reducing symptoms of traumatic stress have been initiated. A randomized controlled trial (RCT) examining the efficacy of the “SUPPORT Coach,” the Dutch equivalent of PTSD Coach, in reducing traumatic stress symptoms in trauma survivors is also ongoing (trialregister.nl).

Another mobile app in the field of PTSD is the “PE Coach,” which has been developed as a tool to support prolonged exposure (PE) therapy and is used concurrently with psychotherapy to support the implementation, dissemination, and patient/provider adherence to PE (Reger, et al., 2013). Research is still needed to test whether PE Coach is useful and effective.

EMDR, by now an accepted and effective treatment for PTSD (Bisson et al., 2013; Forbes et al., 2010) and used on a large scale, might potentially benefit from an m-Health app in terms of treatment efficiency. Questions to be addressed would include whether a task taxing working memory while processing the traumatic memory could be delivered using a mobile app, whether this would affect vividness of the memory (Engelhard, Van Uijen, & Van den Hout, 2010), and in the end whether it could increase the efficiency of treatment.

## Challenges in m-Health and research recommendations

Although there are many arguments in favor of m-Health (Table 1), and researchers and clinicians have certainly started to adopt m-Health initiatives, many challenges remain (Table 2). These issues are listed here accompanied by research questions that address these challenges.

**Table 2:** Checklist m-Health tools development and research

Development phase:
□ having a multidisciplinary team (mental health professionals and IT)
□ based on evidence-based on informed principles or guidelines
□ based on valid and reliable screening and assessment instruments
□ instruments open access or copyright free
□ short and smart
□ providing a source of content
□ providing scientific references
□ tools tested on different devices
□ design matching specific target group and problem
Feasibility and acceptability testing on:
□ reaching the target group
□ user satisfaction, interest, willingness to use
□ perception of treatment credibility
□ expectancies and attitudes
□ adherence to treatment
□ concerns about user privacy, confidentiality and online security
Evaluation of intervention tools:
□ evaluated on efficacy or effectiveness
□ evaluated on cost-effectiveness
□ safety evaluated
□ post-marketing surveillance

### Will the app be used?

In 2015 there will be 500 million smartphone users who will be using a health care app (www.research2guidance.com/). Although the use of smartphones is extremely high and rapidly growing, still some factors may predict ownership of m-Health devices in specific groups, for example, young people have more access than the elderly (e.g., Erbes et al., 2014). For PTSD Coach neither age nor absence of current smartphone use was found to be a barrier in using the app (Kuhn, Greene, et al., 2014). This is encouraging with an aging population not only of veterans but also of civilians. More work is needed to study smartphone use and access to internet in low-income countries and in specific target groups (e.g., those with severe mental health disorders).

It is tempting for the clinician or the developer to be seduced by the state-of-the art IT technology, but in the end the user or patient has to be willing to use the app. End-user needs should therefore be central from the development phase onward.

Research on new apps should involve testing feasibility and acceptability, (i.e., perceived usefulness, user satisfaction, interest, willingness to use, expectancies, and attitudes concerning the mobile app). With apps being used increasingly by an aging population consideration should also be given to matching the design and content of the app with different age groups. Similar issues apply with cultural acceptability. It will be important to know whether innovative strategies increase uptake, for example, adding gameplay, embedding it in a blended care context, and targeting high-risk individuals. Both from a research and an implementation perspective it is extremely interesting but at the same time quite challenging to collect individual data regarding usage of (the different elements of) the app.

### Confidentiality and security

Using data collected by mobile apps to inform health personnel or policy makers also touches on issues like who has access to the data and who owns the data? This is a legal domain that is being dealt with differently in different countries but may have important consequences for the perception of confidentiality and security.

Negative views about computerized self-help intervention regarding user privacy and confidentiality have been expressed; these raise concerns about the likelihood of use (Musiat, Goldstone, & Tarrier, 2014). The concern about security is a potential factor blocking the widespread acceptability and use of m-Health apps. Online security is particularly important when dealing with mental health data. User privacy and confidentiality need to be protected; this involves appropriate collection and handling of user data (including sending data in encrypted format), no unauthorized access, and safe storage of the material collected.

There seems to be a role for researchers, legal experts, and policy makers alike to improve the public perception of m-Health interventions to increase credibility, privacy, and confidentiality.

### Efficacy and innovative designs

In particular, for applications that claim to improve mental health, rigorous evaluation is essential. But with the rapid developments in m-Health, and the constant release of new apps or new versions of apps, research should ideally be quick and affordable and at the same time be able to validate safety; measure quality of care, clinical and cost-effectiveness; and determine whether the app (stand-alone or as an add-on to regular care) is acceptable and safe in comparison with regular face-to-face mental health care. The RCT is still the method of choice for evaluating health interventions.

Fortunately, several certification and vetting initiatives are being established [e.g., onlinehulpstempel.nl: or mirro.nl (Dutch)] and there are also platforms (like Quli.nl) that help to feature evidence-based apps and peer review of mobile apps has been suggested (e.g., imedicalapps.com).

Pilot studies, case–control designs, and alternative (non-randomized) designs to the classical RCT have been proposed (e.g., Sanson-Fisher, D'Este, Carey, Noble, & Paul, 2014); these include multiple baseline testing and interrupted time series. One of the advantages of m-technology is the possibility to collect and aggregate data from large numbers of people anonymously and to look at effect sizes. This information can be fed back to the individual to improve individual prognostic values. Also, interesting new adaptive individualized treatment designs might provide a way to operationalize the strategies leading to personalized treatment (e.g., Almirall, Nahum-Shani, Sherwood, & Murphy, 2014), although evaluation of the optimized treatment versus a control condition would still require a subsequent RCT.

Considering all above, as yet none of the alternatives to the RCT seem to provide the answer to questions of efficacy for m-Health tools; indeed they may produce biased evidence.

More affordable RCT approaches have been proposed, for example, using the web to deliver the health intervention in combination with telephone-based recruitment (Ruggiero et al., 2012); this is particularly interesting for population-based studies recruiting high-risk populations, for example, after disasters and to provide secondary prevention.

In our ongoing study on the SUPPORT Coach (trialregister.nl), we have combined the classical RCT with an innovative and efficient approach using mobile apps both for assessment (SAM, see above) and for the delivery of the intervention. However, because SUPPORT Coach was treated as a medical device by the institutional ethical review board, and because it is being tested in high-risk individuals (screening positive for mild traumatic stress symptoms), we still have to obtain personal, labor-intensive, and expensive, informed consent from each study participant.

Exciting new analyses methods may be helpful when doing research on small sample sizes or on small subsamples. Problems with power and biased parameters can be reduced by including prior information in Bayesian analyses (Van de Schoot, 2015a, 2015b; Van de Schoot, Broere, Perryck, Zondervan-Zwijnenburg, & Van Loey, 2015), compared to using the default method (i.e., maximum likelihood). In our preventive internet intervention study (Mouthaan et al., 2013) we applied latent growth mixture modelling to explore possible latent subgroups within the groups using a Bayesian estimator. In this way, we successfully demonstrated a significant decrease in PTSD symptoms in a small subgroup of patients with severe initial symptoms that with classical methods would have gone undetected.

In sum, there is an academic challenge in dealing with technology in health interventions research. Clear evidence-based guidance is needed on appropriate research designs and analysis methods for evaluation of these technologies that may overcome some of the public and ethical challenges (e.g., equity, availability) and the marketing driven wish to have mobile apps in the app store rather yesterday than tomorrow. Research should take place before releasing apps on the market, but once released, post-marketing surveillance and follow-up is also warranted.

### Cost-effectiveness

The potentially lower cost of m-Health is particularly attractive at current times where increasing (mental) health care expenditure is a concern. e-Health and the use of ICT supporting or improving health care has attracted the interest of governments and health care policy makers who are convinced that the regular use of e-Health applications will lead to affordable, accessible, and high-quality care with more autonomy for patients and who are therefore stimulating e-Health innovations in daily health care ranging from prevention to well-being and treatment, for example, Dutch Action Plan E-Health [Netherlands Organisation for Health Research and Development (ZonMw.nl); or European Commission Horizon 2020 calls].

Alongside cost-effectiveness, Budget Impact Analyses (BIA) are more and more required for mental health care evaluations. It addresses the expected changes in the expenditure of the health care system after the adoption of a new intervention (for details on methods, see Sullivan et al., 2014).

This makes it all the more important that there should be rigorous research on clinical and cost-effectiveness before any large-scale mobile mental health policy is implemented.

### Improving adherence

Some apps are stand-alone applications intended to improve self-management of symptoms, others are used as an add-on to regular evidence-based therapy, or allow or need some degree of therapist guidance, either face-to-face or remotely (e.g., by telephone, email, or text messages).

The anonymity that m-Health tools offer may be an advantage with regard to stigma or shame; the downside may be the lack of warm human in-person support, which we know is beneficial in prevention and recovering from trauma (e.g., Olff, 2012). It is still unclear whether the m-Health alternative forms of support have similar effects as face-to-face contact.

Offering some form of guidance may, however, increase retention and thus effectiveness. Patients have perceived personal feedback and support as positive, which has helped to optimally use the program and to keep them motivated (e.g., Bendelin et al., 2011). This may also take away the concern that some patients—but also clinicians—have about the lack of personal contact when using m-Health tools.

Research questions to address are whether therapist guidance is necessary, and if so, how much as well as how it should be delivered (face-to-face or remotely by telephone, email, or text messages)? Any investigation of the delivery of therapist guidance should also consider how best to motivate clinicians to use or promote m-Health tools.

### Adverse reactions

We know little about adverse reactions to mobile health tools. The US Food and Drug Administration (FDA) has issued guidance in order to regulate a subset of mobile medical mental health applications. Mental health mobile apps may be considered as medical devices and the guidance applies a similar risk-based approach to assure safety and effectiveness of mobile apps as other medical devices. For research this means study protocols have to be submitted to thorough medical ethical review.

Little is known about differential effects in subgroups. Self-help tools will potentially reach more vulnerable patient groups, such as those with a history of complex trauma and severe trauma related disorders. Just as in regular treatment, it may be that patients not having a sufficiently strong emotion regulation capacity may need some skills training in order to effectively optimize the benefits of being exposed to traumatic memories through m-Health tools. Although trauma-focused treatments have been found to be more effective than non-trauma-focused treatments (Bisson et al., 2013), in patients with “Complex PTSD,” interventions that specifically focus on improving emotion regulation and other aspects of functioning preceding trauma-focused work may be of particular benefit (Cloitre, 2015). There is much debate about this in regular face-to-face treatment (Jongh & Broeke, 2014) and in m-Health the research has yet to begin.

### Business models and multidisciplinary collaboration

Although academics and clinicians are increasingly developing m-Health tools, most are mainly interested in creating effective interventions or assessment tools, and having m-Health tools available open access and free of charge. They are typically not thinking in terms of business models, successful products to market, or of building companies (Miron-Shatz, Shatz, Becker, Patel, & Eysenbach, 2014). However, there is a cost to the development and maintenance of mobile apps, as well as the continued updating and improvements that are required. Also, there is a growing demand, even from research grant suppliers (including the European Commission), that products should be exploited and business models developed.

The difference in interests between academia/clinicians and business may also lead to misunderstandings during the development phase when mental health care specialists have to work closely together with IT partners. From ideas to specifications and finally a fully functioning app usually takes several iterations and miscommunications may challenge a tight planning schedule (e.g., due to research grants). New types of potentially sensitive discussions may occur, for instance, on intellectual property (IP), IP rights (IPR), background versus foreground knowledge (created by the project), publication rights, and division of potential future revenues. All these issues needs to be negotiated and documented with all parties involved at the start of development of the app. Ideally, expert centers with multidisciplinary teams should be created with individuals speaking the same “language.”

## An exciting future ahead

Innovative solutions for preventing and reducing traumatic stress symptoms are being developed and valid, tailor-made, easily accessible, and low-cost m-Health tools may contribute to preventing traumatic stress symptoms or posttrauma disorders. The explosive growth of mobile mental health tools on the market has the potential to be an efficient (cost-effective) approach, reducing wait lists and serving a considerable portion of people globally (g-Health). It may increase mental health availability and equity worldwide. With rapid technological developments m-Health may even be outdated soon, but the principles and challenges will also apply to a next generation of high-tech virtual reality or wrist-based wearable devices where the individual may combine trauma exposure exercises with their workout app while getting feedback on heart rate responses or stress hormones. Big data (i.e., individual data at an aggregated level) will feed into this process such that decision support systems will lead the individual to the right clinical pathway, but, as argued for in this paper, hopefully none of this without rigorous research. In the future, apps may need a disclaimer if they have not been validated with scientific research. Table 2 provides a checklist that may guide researchers and all involved in development, qualitative, and quantitative evaluation of the apps.

With this paper I hope to have shown the added value of proper research on m-Health tools and to have given some guidance on important issues in this fast-developing field. Ideally, there should be a joint call from health authorities, academia, research institutes, and the IT sector, as well as the end-users, to examine m-Health before releasing them into the market.

## Supplementary Material

Mobile mental health: a challenging research agenda

Mobile mental health: a challenging research agenda

Mobile mental health: a challenging research agenda

Mobile mental health: a challenging research agenda

Mobile mental health: a challenging research agenda

Mobile mental health: a challenging research agenda

Mobile mental health: a challenging research agenda
