# Supplementary material for: Mobile mental health: a challenging research agenda
Source: Eur J Psychotraumatol. 2015 May 19;6:10.3402/ejpt.v6.27882. doi: 10.3402/ejpt.v6.27882 (PMC4439418; doi:10.3402/ejpt.v6.27882)
Supplement: Mobile mental health: a challenging research agenda [file EJPT-6-27882-s001.pdf]

Mobile mental health: a challenging research agenda

Miranda Olff

Le domaine de la santé mobile (”m-Health”) évolue rapidement et il y existe une croissance explosive des outils psychologiques sur le marché. Le développement passionnant des nouvelles technologies, qui identifient les symptômes, aident les personnes à gérer leur propre santé mentale, encouragent la recherche d'aide, et fournissent à la fois des interventions préventives et thérapeutiques. Ce développement a le potentiel pour être une approche efficace (rentable), réduisant les listes d'attente et pouvant être utilisée par une partie considérable de personnes dans le monde ("g-Health").
Cependant, peu de ces applications mobiles (apps) ont été rigoureusement évaluées. Il y existe peu d'information sur la façon dont les outils de dépistage et d'évaluation sont valides, lesquelles de ces applications mobiles d'intervention sont efficaces, ou de quelles façons les applications mobiles se comparent à des traitements en face-à-face. Mais comment estil possible d’avoir une évaluation scientifique rigoureuse face à la demande croissante - des décideurs politiques, partenaires commerciaux et utilisateurs - pour leur commercialisation rapide?
Dans cet article, l'évolution des outils de ”m-Health” - ciblant le dépistage, l'évaluation, la prévention et le traitement - est examinée par des exemples tirés du domaine de la traumatologie et de l’ESPT. Les difficultés académiques dans l'élaboration et l'évaluation d'outils de ”m-Health” sont abordés. Des orientations fondées sur des preuves sont nécessaires sur les plans de recherche appropriés qui peuvent surmonter certains des défis publics et éthiques (par exemple équité, disponibilité) et le souhait conduit par le marché à avoir des applications mobiles dans le «App Store» hier plutôt que demain.

Mots-clés: e-Health; santé mobile, m-Health; smartphone; applications mobiles / applications; santé mondiale; traumatisme; ESPT; dépression; intervention internet

Citation: European Journal of Psychotraumatology 2015, **6**: 27882 - http://dx.doi.org/10.3402/ejpt.v6.27882
