# Supplementary material for: Mobile mental health: a challenging research agenda
Source: Eur J Psychotraumatol. 2015 May 19;6:10.3402/ejpt.v6.27882. doi: 10.3402/ejpt.v6.27882 (PMC4439418; doi:10.3402/ejpt.v6.27882)
Supplement: Mobile mental health: a challenging research agenda [file EJPT-6-27882-s002.pdf]

Mobile psychische Gesundheit: eine herausfordernde Forschungsagenda

Miranda Olff

Das Feld der mobilen Gesundheit ('m-Health') entwickelt sich sehr schnell und der Zuwachs explosive psychologischer Werkzeuge am Markt scheint zu explodieren. Spannende High-Tech Entwicklungen, die Symptome identifizieren, helfen Betroffenen ihre eigene psychische Gesundheit zu managen, ermutigen dazu, sich Hilfe zu holen und stellen präventive und kurative Interventionen zur Verfügung. Diese Entwicklung hat das Potential ein effizienter Zugang hinsichtlich der Kosten zu werden, Wartelisten zu verkürzen und einer beträchtlichen Anzahl an Menschen global zu helfen („g-health). Dennoch sind bislang nur wenige mobile Applikationen (apps) streng evaluiert worden. Es gibt wenig Informationen wie valide die Screenings und die diagnostischen Instrumente sind, welche Apps effektiv sind, oder wie gut die apps im Vergleich von face-to-face Interventionen abschneiden. Aber wie machbar ist eine strenge wissenschaftliche Evaluation bei steigender Nachfrage, z. B. von Policy-Machern, Geschäftspartnern und Anwendern?
In diesem Artikel werden die Entwicklung von m-Health Instrumenten, die auf Screening, Diagnostik, Prävention und Behandlung abzielen, vorgestellt und diskutiert, insbesondere im Bereich von Trauma und PTBS. Die wissenschaftlichen Herausforderungen in der Entwicklung und Evaluation derartiger Instrumente werden vorgestellt. Evidenz-basierte Orientierung ist erforderlich für angemessene Forschungsdesigns, die einige öffentliche und ethische Herausforderungen (z. B. Gerechtigkeit und Verfügbarkeit) sowie den Wunsch des Marktes mobile apps im App-Geschäft eher gestern als heute zu haben, überwinden.

Schlüsselwörter: e-Health, mobile Gesundheit, m-health, Smartphone, mobile apps/Applikationen, globale Gesundheit, Trauma, PTBS, Depressionen, Internet-Interventionen

Citation: European Journal of Psychotraumatology 2015, **6**: 27882 - http://dx.doi.org/10.3402/ejpt.v6.27882
