# Supplementary material for: Mobile mental health: a challenging research agenda
Source: Eur J Psychotraumatol. 2015 May 19;6:10.3402/ejpt.v6.27882. doi: 10.3402/ejpt.v6.27882 (PMC4439418; doi:10.3402/ejpt.v6.27882)
Supplement: Mobile mental health: a challenging research agenda [file EJPT-6-27882-s003.pdf]

Mobiltelefonos mentális egészség: kihívások a kutatásban

Miranda Olff

A mobil egészség (m-egészség) területe rohamosan fejlődik és számos pszichológiai eszköz van a piacon. Izgalmas technikai vívmányok, amelyek segítenek a tünetek felismerésében, segítik a személyeket a saját mentális egészségük nyomon követésében, támogatják a segítségkérést és más prevenciós és terápiás intervenciót ajánlanak. Az m-egészség lehetőséget nyújt egy hatékony (költséghatékony), globális ellátáshoz (g-egészség), amely a várólisták hosszát is csökkenti.
Ennek ellenére kevés mobil applikációt (app) vizsgáltak meg és értékeltek ki. Kevés információnk van arról, hogy mennyire validak az appos szűrő- és mérőeszközök, illetve mennyiben hasonlíthatók a mobilos intervenciók a személyes kezelésekhez. De vajon mennyire megvalósítható a tudományos kiértékelése ezeknek a programoknak, amikor nagy a nyomás az azonnali forgalomba hozatalért a törvényhozó szervek, üzleti érdekeltségek és felhasználók részéről?
Jelen tanulmányban, az m-egészség legújabb fejlesztései kerülnek bemutatásra – szűrés, kivizsgálás, prevenció, terápia – különös tekintettel a trauma és PTSD területére. Az m-egészség fejlesztése és kiértékelése kerül górcső alá tudományos szempontból. Bizonyítékon alapuló ajánlások szükségesek a megfelelő kutatástervezéshez, hogy kiküszöböljük a különböző kihívásokat (pl. etika, hozzáférhetőség) és a piac kívánságának is eleget tegyünk, hogy az appok ott legyenek minél előbb az „App Store”-ban.

Kulcsszavak: e-egészség; mobil-egészség; m-egészség; okos telefon; mobil applikáció; globális egészség; trauma; PTSD; depresszió; internetes intervenció

Citation: European Journal of Psychotraumatology 2015, **6**: 27882 - http://dx.doi.org/10.3402/ejpt.v6.27882
