# Supplementary material for: Mobile mental health: a challenging research agenda
Source: Eur J Psychotraumatol. 2015 May 19;6:10.3402/ejpt.v6.27882. doi: 10.3402/ejpt.v6.27882 (PMC4439418; doi:10.3402/ejpt.v6.27882)
Supplement: Mobile mental health: a challenging research agenda [file EJPT-6-27882-s004.pdf]

Salud mental móvil: Un programa de investigación difícil

Miranda Olff

El campo de la salud móvil ('salud-m') se está desarrollando rápidamente y hay un crecimiento explosivo de herramientas psicológicas en el mercado. Existen descubrimientos de alta tecnología nuevos y emocionantes, que identifican síntomas, ayudan a los individuos a gestionar su propia salud mental, animan a buscar ayuda y aportan intervenciones tanto preventivas como terapéuticas. Este descubrimiento tiene el potencial de ser un abordaje eficaz (rentable) para reducir listas de espera y servir a una cantidad considerable de personas a nivel global ("salud-g").

Sin embargo, pocas aplicaciones móviles han sido rigurosamente evaluadas. Hay poca información sobre lo válidas que son como herramientas de filtrado y evaluación, qué aplicaciones de intervención móvil son eficaces y cómo se comparan las aplicaciones móviles frente a los tratamientos cara a cara. Pero ¿cuán viable es una evaluación científica rigurosa con el incremento de la demanda, desde políticos, socios de negocios y usuarios, dado lo rápido que salen al mercado?

En este artículo, y con ejemplos del campo del trauma y del TEPT, se revisan los descubrimientos de las herramientas de salud-m centradas en el filtrado, la evaluación, la prevención y el tratamiento. Se le está prestando atención a los retos académicos relacionados con el desarrollo y la evaluación de herramientas de salud-m. Es necesaria una guía basada en la evidencia sobre diseños adecuados de investigación que puedan superar algunos de los retos públicos y éticos (p.ej. equidad, disponibilidad) y el deseo del mercado de que haya aplicaciones móviles en el "*App Store*" para ayer mejor que para mañana.

Palabras clave: salud electrónica; salud móvil, salud-m; teléfono inteligente; aplicaciones móviles; salud global; trauma; TEPT; depresión; intervención por internet

Citation: European Journal of Psychotraumatology 2015, **6**: 27882 - http://dx.doi.org/10.3402/ejpt.v6.27882
