# Supplementary material for: Mobile mental health: a challenging research agenda
Source: Eur J Psychotraumatol. 2015 May 19;6:10.3402/ejpt.v6.27882. doi: 10.3402/ejpt.v6.27882 (PMC4439418; doi:10.3402/ejpt.v6.27882)
Supplement: Mobile mental health: a challenging research agenda [file EJPT-6-27882-s005.pdf]

Mobil mental sağlık: Zorlayıcı bir araştırma gündemi

Miranda Olff

Mobil sağlık ('m-Sağlık') alanı çok hızlıca gelişmekte ve market üzerindeki psikolojik araçlarda büyük bir gelişme mevcuttur. Heyecan verici ileri teknolojik gelişmeler, semptomları tanımlayan, bireylere kendi mental sağlıklarını yönetmeye yardım etmekte, yardım aramaları için desteklemekte ve hem önleyici hem de teröpatik müdahaleler sağlamaktadır. Bu gelişim etkili (uygun maliyet) bir yaklaşım olabilecek, bekleme listesini azaltabilecek ve küresel olarak ("g-Sağlık") insanların gözle görülür şekilde büyük çoğunluğuna hizmet verebilecek potansiyele sahiptir. Buna rağmen, mobil uygulamaların (apps) az bir kısmı önemli şekilde değerlendirilmiştir. Geçerli tarama ve değerlendirme araçlarının nasıl olduğu, mobil müdahale uygulamalarının hangilerinin etkili olduğu, ya da yüz yüze görüşmelere kıyasla mobil uygulamaların ne derece iyi olduğuna dair çok az bilgi bulunmaktadır. Fakat politika oluşturucular, iş ortakları ve kullanıcılar tarafından gelen, bunların çabuk piyasaya sürülmense yönelik, artan taleplerle birlikte titiz bilimsel değerlendirme kadar mümkündür?
Bu yazıda, taramaya, değerlendirmeye, önlemeye ve tedaviye yönelik mobil sağlık araçlarının gelişimi travma ve TSSB alanından örneklerle gözden geçirilmiştir. Mobil sağlık araçlarının gelişimi ve değerlendirilmesindeki akademik zorluklar ele alınmaktadır. Toplum ve etik zorlukların (örn. eşitlik, kullanılabilirlik) ve yarından ziyade dünkü "App Store" daki olmasına yönelik market odaklı dileklerin bazılarının üstesinden gelebilecek uygun araştırma dizaynları üzerine kanıt temelli yönlendirmeye ihtiyaç duyulmaktadır.

Anahtar Kelimeler: e-Sağlık; mobil sağlık; m-Sağlık; akıllı telefon; mobil apps/ uygulamalar; küresel sağlık; travma; TSSB; depresyon; internet müdahale

Citation: European Journal of Psychotraumatology 2015, **6**: 27882 - http://dx.doi.org/10.3402/ejpt.v6.27882
