# Supplementary material for: Mobile mental health: a challenging research agenda
Source: Eur J Psychotraumatol. 2015 May 19;6:10.3402/ejpt.v6.27882. doi: 10.3402/ejpt.v6.27882 (PMC4439418; doi:10.3402/ejpt.v6.27882)
Supplement: Mobile mental health: a challenging research agenda [file EJPT-6-27882-s006.pdf]

Mobiele geestelijke gezondheidszorg: een uitdagende onderzoeksagenda

Miranda Olff

Het veld van de gezondheidszorg waarin van mobiele applicaties (apps) gebruik gemaakt wordt (m-Health) verandert snel en er is een explosieve groei van allerlei psychologische hulpmiddelen op de markt. Spannende hightech ontwikkelingen maken het al mogelijk dat symptomen snel geïdentificeerd worden, dat mensen zelf aan hun geestelijke gezondheid kunnen werken, sneller hulp zoeken als dat nodig is, en er zijn apps voor preventieve interventies en behandeling. De m-Health ontwikkelingen zijn potentieel een efficiënte kosten-effectieve benadering die wachtlijsten kan verminderen en bovendien een aanzienlijk deel van de bevolking wereldwijd kan bedienen (g-Health).
Helaas zijn er nog maar weinig mobiele apps grondig wetenschappelijk geëvalueerd. Er is weinig informatie over hoe valide screenings- en meetinstrumenten zijn, welke interventie apps effectief zijn, of over hoe mobiele apps zich verhouden tot de behandeling in de spreekkamer. Maar is het wel haalbaar serieus wetenschappelijk onderzoek te doen naar deze apps terwijl er tegelijkertijd een groeiende vraag is naar het snel beschikbaar komen van de apps vanuit de politiek, de zorg, de zakelijke partners, en ook de gebruikers?
In dit artikel worden de ontwikkelingen in de m-Health beschreven op het gebied van screening, diagnostiek, preventie en behandeling rondom trauma en PTSS. De uitdagingen die er liggen tijdens de ontwikkeling en de evaluatie van apps worden besproken en concrete checklists worden aangereikt. Wetenschappelijke evidence-based richtlijnen zijn nodig voor goed onderzoek naar mobiele apps, om beter om te kunnen gaan met publieke en morele issues (zoals beschikbaarheid en toegankelijkheid van zorg voor iedereen), en handvatten geboden worden aan de door de markt gedreven wens de apps liever gisteren dan vandaag in de “App Store” beschikbaar te hebben.

Keywords: e-Health; mobiele gezondheid; m-Health; smartphone; mobiele apps/applicaties; wereldwijde gezondheidszorg; trauma; PTSS; depressie; internet intervention

Verantwoordelijke editor: Marylene Cloitre, National Center for PTSD in Palo Alto, CA, USA.

Citatie: Olff, M. (2015). Mobile mental health: A challenging research agenda. European Journal of Psychotraumatology, 6, 27882, doi: http://dx.doi.org/10.3402/ejpt.v6.27882
