# Supplementary material for: Mobile mental health: a challenging research agenda
Source: Eur J Psychotraumatol. 2015 May 19;6:10.3402/ejpt.v6.27882. doi: 10.3402/ejpt.v6.27882 (PMC4439418; doi:10.3402/ejpt.v6.27882)
Supplement: Mobile mental health: a challenging research agenda [file EJPT-6-27882-s007.pdf]

Mobilno mentalno zdravlje: izazov istraživačkom planu

Miranda Olff

Polje mobilnog zdravstva ("m-zdravstvo") se brzo razvija i postoji eksplozivan rast psiholoških alata na tržištu. Uzbudljiv razvoj visokih tehnologija, koje prepoznaju simptome, pomaže pojedincima da rukovode svojim mentalnim zdravljem, hrabri ih da traže pomoć, i obezbeđuje i preventivne i terapijske intervencije. Ovakav razvoj ima potencijal da bude efikasan (cost-effective) pristup, redukujući liste čekanja i opslužujići značaj broj ljudi širom sveta ("g-zdravstvo"). Međutim, svega nekoliko mobilnih aplikacija (apps) je strogo kontrolisano. Postoji malo informacija o validnosti alatki za skrining i procenu, koje su od mobilnih apps za intervencije efikasne ili kako se mogu porediti mobilne apps sa licem u lice tretmanima. Ali koliko je izvodljiva stroga naučna evaluacija uz rastuće potrebe – od osiguranja, poslovnih partnera i korisnika- za njihovo brzo aktiviranje?
U ovom su radu pregledno prikazani razvoj alata za m-zdravstvo-ciljanje skrininga, procene, prevencije i tretmana- sa primerima iz oblasti traume i PTSP-a. Naglašeni su akademski izazovi u razvoju i evaluaciji m-zdravstvenih alatki. Smernice na osnovu dokaza su potrebne da bi se prevazišli neki opšti i etički izazovi (na pr. pravičnost, dostupnost) i želja vođena zahtevima tržišta da mobilne apps imamo dostupne u "app prodavnici" juče radije nego sutra.

Ključne reči: e-zdravstvo;, m-zdravstvo; mobilne apps/aplikacije;globalno zdravlje; PTSP; depresija; intervencije inetrnetom

Citation: European Journal of Psychotraumatology 2015, **6**: 27882 - http://dx.doi.org/10.3402/ejpt.v6.27882
